# Influence of the Gut Microbiota on the Development of Neurodegenerative Diseases

**DOI:** 10.1155/2022/3300903

**Published:** 2022-09-30

**Authors:** Mahendra P. Singh, Riya Chakrabarty, Shabnam Shabir, Sumaira Yousuf, Ahmad A. Obaid, Mahmoud Moustafa, Mohammed Al-Shehri, Ahmed Al-Emam, Abdulhakeem S. Alamri, Walaa F. Alsanie, Majid Alhomrani, Anastasiia D. Shkodina, Sandeep K. Singh

**Affiliations:** ^1^School of Bioengineering and Biosciences, Lovely Professional University, Jalandhar-Ludhiana GT Road, Phagwara, 144411 Punjab, India; ^2^Laboratory Medicine Department, Faculty of Applied Medical Sciences, Umm Al-Qura University, Makkah, Saudi Arabia; ^3^Department of Biology, College of Science, King Khalid University, 9004 Abha, Saudi Arabia; ^4^Department of Botany and Microbiology, Faculty of Science, South Valley University, Qena, Egypt; ^5^Department of Pathology, College of Medicine, King Khalid University, Abha, Saudi Arabia; ^6^Department of Forensic Medicine and Clinical Toxicology, Faculty of Medicine, Mansoura University, Mansoura, Egypt; ^7^Department of Clinical Laboratory Sciences, the Faculty of Applied Medical Sciences, Taif University, Taif, Saudi Arabia; ^8^Centre of Biomedical Sciences Research (CBSR), Deanship of Scientific Research, Taif University, Saudi Arabia; ^9^Department of Neurological Diseases, Poltava State Medical University, 36000 Poltava, Ukraine; ^10^Indian Scientific Education and Technology Foundation, 226002, Lucknow, India

## Abstract

Neurodegenerative disorders are marked by neuronal death over time, causing a variety of cognitive and motor dysfunctions. Protein misfolding, neuroinflammation, and mitochondrial and protein clearance system dysfunction have all been identified as common pathways leading to neurodegeneration in recent decades. An altered microbiome of the gut, which is considered to play a central role in diseases as well as health, has recently been identified as another potential feature seen in neurodegenerative disorders. An array of microbial molecules that are released in the digestive tract may mediate gut-brain connections and permeate many organ systems, including the nervous system. Furthermore, recent findings from clinical as well as preclinical trials suggest that the microbiota of the gut plays a critical part in gut-brain interplay and that a misbalance in the composition of the gut microbiome may be linked to the etiology of neurological disorders (majorly neurodegenerative health problems); the underlying mechanism of which is still unknown. The review aims to consider the association between the microbiota of the gut and neurodegenerative disorders, as well as to add to our understanding of the significance of the gut microbiome in neurodegeneration and the mechanisms that underlie it. Knowing the mechanisms behind the gut microbiome's role and abundance will provide us with new insights that could lead to novel therapeutic strategies.

## 1. Introduction

Before the 1990s, the concept of microbiome or gut-microbiota was unclear; therefore, only cultivable bacteria were known to people. The literature reveals that only approximately 30% of the entire gut microbiome is known [1]. With the advancement in technology, where genome sequencing and associated technologies are gaining rapid pace, deciphering their roles on both physiological and pathological grounds has been elucidated. Furthermore, an entire length of 16S rRNA can be easily sequenced with Third Generation Sequencers such as PacBio Sequel. This enables us to identify various species in a collective sample. In addition, met genomics aids in unveiling the genome collected from the surroundings, which later enhances our acquaintance regarding the associative relationship of the microbiota with that of the health of an individual [2].

Progressive studies have established that the development of neurodegeneration has its strings attached to the gut-microbiome. Several structures have been identified to contribute to the establishment of cross-talk between the two [3]. Researchers have revealed that the conformation of the microbiome gut can be disturbed through a variety of medical, physiological, and environmental stimuli. Among them, antibiotic exposure to the gut serves to be of prime importance. In the following text, we will discuss the microbiome and its influence on neurodegeneration.

Studies on axenic mice, scientifically termed the “gnotobiotic” murine model, reveal that gut bacteria is a prerequisite for the proper performance of the digestive tract. Moreover, it has also been proven to possess a major significance in the sustenance of the accomplishment capability of enteric nervous system (ENS), the functioning of which is independent of the central nervous system (CNS). The neuronal circuit of the ENS is interpenetrated into the wall of the gut; moreover, the neurons associated with it are of the motor type, sensory type, and interneuron type [4].

## 2. Knowing the Gut Microbiota

The microbiota is omnipresent and found at a differential location on or within the human being. These locations extensively include the cavities of the body such as the buccal cavity, inside the acoustic meatus, nasopharyngeal cavity, digestive tract, urogenital passage, and obviously on the surface of the epidermal layer [5]. The digestive tract is also known as the GI tract predominating, in the quantity of the microbiome it contains, which accounts for approximately 95% of the summate of microbes in the living body. The literature demonstrates that the digestive tract houses approximately 100 trillion bacteria in addition to a large number of discrete bacterial variants [6]. It is presumed that more than 98% of the bacterial ecosystem in the alimentary canal consists of aerophilic microbes, which are also referred to as aerobic bacteria, and the remaining 1% peculiarly contains archaebacteria, fungi, and protozoans. In addition to the variety of microorganisms, studies have revealed that two stellar bacterial phyla predominantly constitute the gut-microbiome of human beings: Firmicutes and Bacteroidetes [7, 8]. Studies further reveal that Firmicutes constitute approximately 35-80% of the microbiome, while Bacteroidetes constitute approximately 17-60% of the gut microbiome [9]. Amusingly, obesity is also found to be related to the ratio of Bacteroidetes to Firmicutes to some extent [10]. Further comprehensive efforts in the field of research towards the microbiome of the gut illustrated that the settlement of microbes inside the gut is initiated at birth and is completely achieved in the upcoming three years of birth [11].

## 3. Neurodegeneration and Associated Diseases

Neurodegeneration, as the name implies, refers to the degeneration of neurons. Although it can occur for a variety of reasons, it has been observed to occur more frequently in older people, such as the ability of cell renewal and repair declines.

### 3.1. Parkinson's Disease (PD)

**Figure 1:**
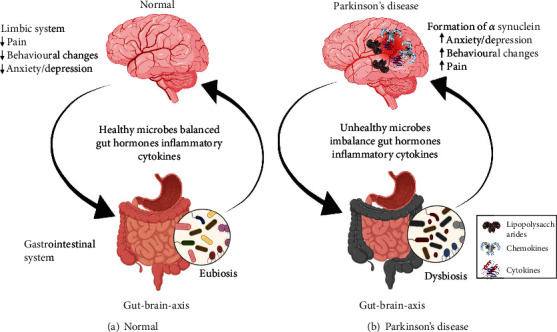
An illustration of microbiome-gut-brain axis in Parkinson's condition. The blood-brain barrier (BBB) may be permeable to lipopolysaccharide (LPS) and other bacterial metabolites, which could allow them to enter the brain and cause the release of several chemokines and cytokines that would contribute to inflammation in Parkinson's disease. Leaky gut also known as compromised gut epithelial barrier integrity may result from microorganisms in the lumen of the gut promoting inflammatory mechanisms and harming intestinal enterocytes. Bacterial metabolites, like LPS, can cross the compromised gut barrier from the lumen of the gut to the bloodstream and potentially cause systemic and neuroinflammation in the brain.

Parkinson's disease is mostly witnessed in the elderly population. Investigations have revealed that the degeneration of the dopamine-influenced nigrostriatal pathway gives rise to the malady, which is further accompanied by typically abnormal movements. The involvement of the enteric neuronal system has been established under the influence of *α*-synuclein [12]. Parkinson's disease was previously thought to be an idiopathic disorder with no hereditary basis in the vast number of affected roles. Despite this, a small percentage of the affected people were found to have a hereditary line of the disease, and the transmission of the disorder from one generation to the next was confirmed to be autosomal dominant. Two important missense mutations in the coding of the *α*-synuclein gene have been ascertained by researchers (Figure 1). It has also been ascertained that this particular mutation is liable for the development of the disease [13, 14].

Distorted *α*-syn clusters, also known as Lewy body depositions or deposits, play a role in the degeneration of neurons (dopaminergic) in the part of the brain known as substantia nigra (controls movements) and various other related functions that lead to the onset of several key non-motor and motor characteristics of Parkinson's disease (PD), such as dementia, tremor, bradykinesia, anxiety, digestive tract disability, rigidity, behavioral changes, and postural instability [15]. The concept of a “gut-brain axis,” a bidirectional communication mechanism between the enteric and central nervous systems and the digestive system, has been supported by the discovery of *α*-syn clusters in peripheral locations, including the enteric nervous system (ENS), despite their most frequent location in the brain [16].

The mutation results in the conversion of an alanine residue from *α*-synuclein's 53rd position to threonine, which is also denoted as A53T. The alanine residue at the 30th position of the *α*-synuclein peptide changes to proline in the second mutation, which is also represented as A30P [14]. Further studies have identified that the A53T mutation is a potent cause for the generation of hereditary PD. This discovery led to the fact that *α*-synuclein is the key element of Lewy bodies and Lewy neurites [17]. Lewy bodies are intracytoplasmic inclusion bodies generally localized in the medulla-oblongata and have a circular appearance. It consists of an impenetrable eosinophilia center and a comparatively thin circumferential corona [18].

### 3.2. Alzheimer's Disease (AD)

Alzheimer's disease (AD) is also one of the prevalent variants of dementia around the globe. Alzheimer's disease generally has 2 types: sporadic and familial. Another way of categorizing AD is via the age of onsets of the disorder, such as early onset or late onset. Although the primary cause for the generation and progression of AD is malfunctioning or nonfunctional neurons in specific regions of the encephalon, there are other factors that more or less contribute to the same.

Recognizing physical and behavioral changes of AD-affected roles are sharp depletion of retention, difficulty, and discomfort in speech and writing, the capability of resolving problems reduced considerably, inability to retrace objects placed elsewhere, etc. These symptoms become life-threatening with advancement in the stages of the malady [19].

**Figure 2:**
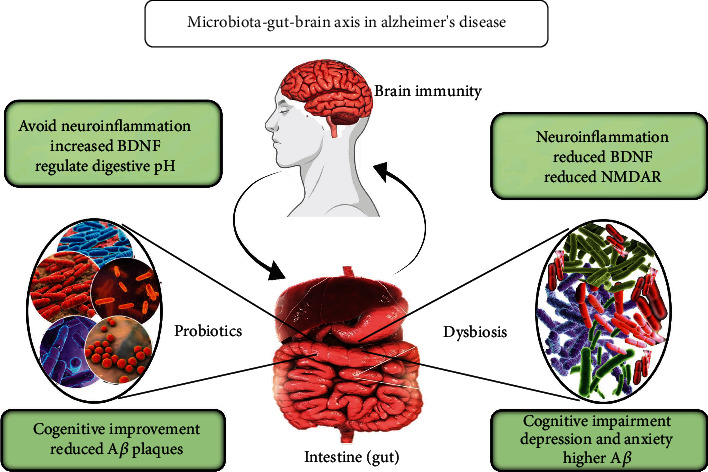
Crosstalk between the gut microbiome and the brain in Alzheimer's disease. Metabolic, endocrine, neurological, and immunological communication routes exist between the gut microbiota and the brain, and they can act independently or collaboratively.

Investigations at the cellular and molecular levels have revealed two proteins that are potentially liable for the generation and progression of neurodegeneration. Researchers have revealed that patients suffering from the malady have an accumulation of amyloid-*β* and *Tau* protein in the tissues of the encephalon [18]. It has been identified that amyloid-*β* accumulates towards the exteriors of the nerve cell. *Tau* protein is modified into a couple of helical filaments that further gather in association with each other to form neurofibrillary tangles [20]. Both the aforementioned are the primary characteristic etiologies of AD (Figure 2).

Furthermore, after oligomerization, the amyloid-*β* readily diffuses into the synaptic cleft. This hinders the transmission of signals from one neuron to another, ultimately rendering the cell nonfunctional [21]. On the other hand, *Tau* protein is involved in the polymerization of tubulin protein in microtubules. Generally, it is a protein associated with 2-3 phosphates per molecule; after hyperphosphorylation, the amount of phosphate abnormally increases to 5-9 phosphates per molecule of *tau* [22]. Such hyperphosphorylated conditions lead to loss of affinity of *tau* towards the tubulin protein, which further affects the construction and maintenance of microtubules [23].

An elevating number of researches point to a bidirectional association between the gut, bacteria, and brain, known as the gut-microbiota-brain axis that may play a significant function in the development of the brain [24]. The microbiome can affect the regulation of the brain-derived neurotrophic factor (BDNF), N-methyl-D-aspartate receptor (NMDAR), and neuroinflammation through direct neural, neuroendocrine, and immunological mechanisms. In AD brains, there were found to be lower levels of BDNF [25]. It is interesting to note that mice lacking BDNF exhibit altered GI-tract innervation development [26]. The hippocampus and cortex of germ-free mice were found to have lower levels of BDNF expression, and it was discovered that lower levels of BDNF expression were specifically linked to higher levels of anxiety and progressive cognitive dysfunction [27].

### 3.3. Amyotrophic Lateral Sclerosis

Amyotrophic lateral sclerosis (ALS) is considered as an exceptional but devastating neuronal toxic condition that kills people after 1 or 3 years of identification. The global incidence of amyotrophic lateral sclerosis is expected to be 1.75 per 100,000 person-years (1.45 for females and 2.03 for males). Amyotrophic lateral sclerosis is among the most difficult diagnoses for clinical practitioners to make and affect due to its debilitating course and current lack of viable treatment. Despite this fact, observational studies have revealed a variety of risk variables. Amyotrophic lateral sclerosis, particularly sporadic ALS, is deemed to occur by a mixture of hereditary as well as non-genetic causes [28–30]. Pathological features of this disease include neuroinflammation, motor neuron loss, axon loss, and cytoplasmic protein aggregation in the vertebral column and motor cortex. Based on genetic and pathophysiological data, as well as clinical observations, various disease pathways for amyotrophic lateral sclerosis have been speculated. Among the most frequently discussed reasons are faulty repair of DNA, defective impaired proteostasis, RNA metabolism, faulty nucleocytoplasmic transport, damaged axonal transport, inflammatory processes, mitochondrial dysfunction, and inflammation of neurons [15]. Several monitoring circuits have been found to monitor immune response in ALS patients. A batch of lymphocytes (T-cells) known as T (Treg) cells reduced the progression of disease in ALS-related animal models and is related to a mild form of ailment in humans. One possible explanation is that FOXP3+ Treg cells in the CNS reduce inflammation caused by microglia and neurotoxic T lymphocytes in ALS, thereby contributing to the CNS's immunological privilege and self-tolerance [31].

During the last 10 years, there has been a striking increase in studies on the human microbiome, or the microscopic creatures that reside in the human body. In comparison to other colonization sites, such as the airway, skin, and urogenital tract, the alimentary canal hosts the majority of the human microbiota. Metabolism and adaptive immunity regulation are two important physiological roles of the microbiome of humans [32]. As a consequence, the microbiota of the gut aids in the protection of a healthy body and, when degraded, in the onset of ailments, either directly through interplay with the epithelial barrier and immune system (mucosal), indirectly through metabolite synthesis, or both. Responsible mechanisms for respective effects could include decreased energy-harvesting capacity, host gene activities in the control of storage of fat, and immunological reactions. In addition to influencing immune reactions and energy metabolism, the microbiota of the gut may show a significant function in neurodegeneration by influencing protein ubiquitination, transmission, and aggregation from the peripheral nervous system to the CNS [27].

**Figure 3:**
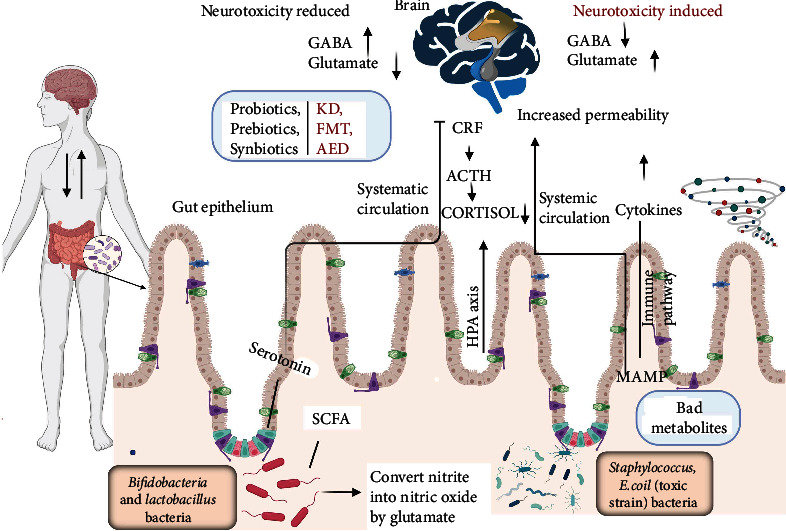
Possible mechanisms responsible for the effect of the digestive tract microbiome on the pathogenesis of ALS. Microbial metabolic end products may indirectly influence the central nervous system (CNS) through immune system modulation. Toxins and neuroactive metabolites produced by a damaged intestinal epithelial barrier or enteric bacteria can overcome the blood-brain barrier, diffuse to the systemic circulation, and impact on ALS pathogenesis. Metabolites produced by bacteria can alter energy homeostasis, encourage oxidative stress, and cause mitochondrial dysfunction and neuroinflammation.

ALS also known as motor neuron disease (MND) has been linked to an imbalance of the neurotransmitter gamma-aminobutyric acid (GABA) and other related modulators. The death of nerve cells (motor neurons) in ALS is thought by scientists to be a result of excessive glutamate exposure [33]. Motor neurons may be protected, and the disease's progression may be slowed by preventing the glutamate level from rising. Immunomodulators and/or cytokines are released by the immune system and microbiome of the gut and may have systemic effects on the host [34]. A variety of neurotransmitters are implicated in the interconnection between the host as well as the gut bacteria, whereas the gut bacteria produce short-chain fatty acids (SCFAs) and other bacterial metabolic products that could have an impact on the brain function. In some cases, the secretion of abnormal physiological levels of ACTH is linked to the loss of motor neurons, motor function, and/or muscle strength with the subsequent manifestation of ALS symptoms [35]. By controlling the hypothalamic pituitary adrenal (HPA) axis in ALS, the corticotropin-releasing factor (CRF) plays a vital part in the stress reactions (Figure 3).

The production of neuroactive metabolites and toxins by microbiota may have a direct impact on the CNS and neuronal health, while immune response, dietary compounds, and drug metabolism may have an indirect impact [36]. For instance, enterochromaffin cells can be directly stimulated by gut microbes and their metabolites, such as SCFAs, to produce several neuropeptides or neurotransmitters, such as serotonin that can diffuse into the bloodstream reach the brain, and affect CNS functions [37]. These neuropeptides and neurotransmitters include peptide YY, neuropeptide Y, and cholecystokinin. The intestinal epithelium controls the transfer of particular bacterial byproducts (such as SCFAs, vitamins, or neurotransmitters) into the bloodstream, which can then spread to the CNS via the circulatory system [38]. This allows metabolites, neuropeptides, and neurotransmitters derived from the circulating microbiota to enter the central nervous system and directly affect its neurobiology.

### 3.4. Pathogenesis of Neurodegenerative Disorders (Oxidative Stress and Neuroinflammation) of Alzheimer's Disease

The aggregation of various specific proteins inside the CNS is comparable to neurodegenerative health conditions like PD, ALS, and AD. Protein misfolding appears to occur in AD, resulting in an accumulation of abnormally folded tau-proteins and beta-amyloid in the neurons. A transmembrane protein called amyloid-precursor protein (APP) is essential for the growth of neurons, survival, and repair (post-injury) under optimum conditions [39]. However, in AD, enzymes break APP into a tiny peptide called A, which are 38-44 amino acids long. Senile plaques are quite dense formations of A that form clusters and accumulate outside neurons. Furthermore, phosphorylation of Tau protein causes abnormal aggregation and instability of this protein in AD. In Parkinson's disease (PD), the protein alpha-synuclein (A-Syn) ties to ubiquitin and forms proteinaceous cytosolic inclusions known as Lewy bodies. Dopaminergic neurons die as a result of uncontrolled growth and post-translational amendment of A-Syn [40]. The gene known as the huntingtin gene (*Htt*), which encodes the protein huntingtin, is present in all humans in two copies (*Htt*). The mutant huntingtin protein (mHtt) is prone to cluster formation. These peptides are retrogradely translocated into the cell body for digestion by lysosomes during the normal clearance process of cells. Under inflammatory conditions, these mutant polypeptides assemble and disrupt the retrograde transit of molecular components that causes damage to microtubules and molecular motors, resulting in cellular damage and leading to chronic neuron degeneration [41].

### 3.5. ROS as a Major Cause of Misfolding of Proteins

There is a link between ROS inequity and neurofibrillary tangles proteins. In comparison to the previously described phenomenon of oxidative stress causing the formation and agglomeration of amyloid plaques, misfolded proteins could also cause excessive ROS, resulting in neurotoxicity. Amyloid-beta formation encourages ROS production, according to various lines of evidence. A causes the pro-oxidative enzyme NADPH-dependent oxidase to be activated, resulting in the generation of O_2_•. Furthermore, H_2_O_2_ is emitted directly during the amyloid-beta aggregation process. By reducing divalent metal ions (Cu_2_+, Fe_2_+), amyloid-beta can transform O_2_ into H_2_O_2_. ROS accumulation exacerbated by amyloid-beta causes lipid peroxidation and the subsequent production of cytotoxic plaques. Amyloid-beta also causes neurotoxicity by disrupting Ca_2_+ homeostasis [42].

### 3.6. Prevention and Therapy of ALS by the Gut Microbiota

Inflammation of neurons known as neuroinflammation is a well-studied ALS pathogenic process marked by a composite dysregulation of both peripheral and resident immune lymphocytes. The principal characteristics are astrocyte and microglia activation, T-cell infiltration, and enhancement of proinflammatory cytokines [43]. The microbiota of humans shows a significant impact on their immunity during growth when lymphocytes learn to distinguish among required dangerous pathogens as well as commensals and later in life when microorganisms help to maintain immunological homeostasis [44, 45]. Pathogen-free mice exhibit numerous problems related to the immune system [46]. The underlying processes that cause the link between the immune system and microbiome are still unknown. Various compounds generated from bacteria are hypothesized to behave as immunomodulators, including aryl hydrocarbon receptor ligands, short-chain fatty acids, polysaccharides, and polyamines [47]. Mice lacking gut microbiota also demonstrate that the microbiota is linked to the CNS via the immune system. In pathogen-free as well as antibiotic-administered murine models, neurodegeneration and immunological disabilities are observed [48].

### 3.7. Prevention of Neurodegenerative Disorders by Short-Chain Fatty Acids (SCFAs)

Short-chain fatty acids (SCFAs) are minute organic monocarboxylic acids with 4 to 6 carbon atoms that are formed inside the intestine by the microbiota during microbial digestion of nondigestible poly-saccharides such as dietary fiber and complex carbohydrates [49]. Although fiber fermentation is the most likely source of SCFAs, amino acid metabolic activity can also produce propionate, acetate, and butyrate. These metabolic pathways are used by only approximately 1% of the microbiome in the colon to produce SCFAs [44]. Fermentation of protein in the intestine tract, where carbs are already diminished, generates potentially dangerous metabolic products such as sulfides, phenols, and ammonia as well as distinct branched-chain fatty acids (BCFA). Moreover, the enzyme butyryl-CoA:acetyl-CoA transferase can convert acetate produced by glycolysis acetyl-CoA into butyrate and bovine milk lipids can also provide butyrate. [50].

SCFA mixtures have been discovered to suppress the generation of inflammatory markers (cytokines), promote T cell proliferation in diverse directions, reinstate functionality maturation of microglia, and cause the phenotype metamorphosis of microglia to reduce inflammation. As a result, the combination of SCFAs plays a fundamental task in the immunological modulation of abnormalities of CNS [51].

Short-chain fatty acids are considered to play a vital role in communication between the microbiota, brain, and gut. In addition to giving energy to cells and regulating microglial maturation, these microbial metabolites play a role in influencing neuronal activity. SCFAs have been shown to affect neurotransmitter and neurotrophic factor levels [52]. Previous studies have demonstrated that acetate increases anorexigenic neuropeptide expression and modifies the extent of the neurotransmitters glutamine, GABA, and glutamate in the hypothalamus. Propionate and butyrate affect intracellular potassium levels, implying that SCFAs are involved in the investigation of cellular signaling pathways [53]. SCFAs (e.g., propionate) regulate sympathetic nervous system activity via interactions with G protein coupled receptors (GPRs), including the GPR-43 and GPR-41 receptors of ENS ganglia. Stimulation of such receptors in sympathomimetic neurons is regulated by signaling pathways such as the G*βγ*–PLC beta–MAPK pathway, which modulates the organism's energy requirement and regulates physiological balance. Changing the quantity of SCFAs and ketone bodies generated from them within the liver, in addition to their ratio, induces changes in energy metabolism and the sympathetic nervous system function. Besides providing energy to the central nervous system's cells, SCFAs including propionate and butyrate impact intracellular potassium levels, implying that SCFAs are involved in the functioning of cellular signaling pathways. Certain SCFAs, specifically, affect the transcription of the gene encoding tryptophan hydroxylase, a crucial enzyme in the serotonin manufacturing pathway, as well as tyrosine hydroxylase, an enzyme that is implicated in the biosynthesis of noradrenaline, dopamine, and adrenaline in their rate-limiting step and hence has an impact on brain neurochemistry [54].

Serotonin is produced primarily in the central nervous system by serotonergic nerve cells in the raphe nuclei. Impaired serotonin expression and activity in the brain have been linked to the pathophysiology of psychiatric conditions such as depression and anxiousness. It is vital to note that roughly 85-90 percent of serotonin is generated inside the human body's peripheral tissues, primarily through specific cells known as enterochromaffin in the epithelium of the intestine. On the other hand, serotonin cannot pass the blood-brain barrier, although its precursor tryptophan can. Enterochromaffin cells in the gut use tryptophan from protein intake as a precursor to producing serotonin, which is controlled by the bacterial kynurenine production pathway. Microbes that form spores (mostly *Clostridia*) in the digestive canal can endorse serotonin biosynthesis by raising the expression of genes of tryp-hydroxylase 1 enzyme (TPH1) (rate-limiting) in colon cells (enterochromaffin cells), and even some metabolic products generated by such bacteria have indeed been distinguished as the molecules that prompt this activity. Staphylococci have also been shown to produce serotonin via decarboxylating the precursor 5-hydroxytryptophan (5-HTP) using Staphylococcal aromatic amino acid decarboxylase (Sada) [55].

Modification in the transcription of cognition-relevant neuromodulators such as the serotonin transporter, neurological neurotrophic factor, receptor subunit 2B (N-methyl-D-aspartate), and Y-neuropeptide system may be associated with this cognitive deficit [56]. Neurotrophic factors such as glial cell line-derived neurotrophic factor (GDNF), nerve growth factor (NGF), and brain-derived neurotrophic factor (BDNF) have been shown to modulate the growth, sustenance, and distinctions of synaptic connections inside the central nervous system (CNS). Short-chain fatty acids have also been linked to improvements in learning and memory, as well as a variety of brain disorders [57]. Short-chain fatty acids also demonstrated improvement in sleep, decrease the action of orexigenic neurons in the hypothalamus that articulate Y-neuropeptide, and alter ghrelin receptor signaling, all of which make a significant contribution to appetite control and circadian rhythm.

### 3.8. SCFAs and Neurodegeneration concerning Alzheimer's Disorder

SCFAs may also influence important neuropathological events underlying AD, according to mounting data. AD is the major prevalent type of neuronal, distinguished by increasing cognitive deficits. Given that Alzheimer's disease has complicated pathogenesis and that appropriate therapies to prevent disease development are still absent, numerous studies have examined the advantages of having a healthy microbiota in reducing AD advancement, as well as the link between dysregulation and progression of the disease. SCFAs inhibit the construction of neurotoxic fibrils, the primary toxins accountable for synaptic disruption and cognitive deficits in AD, by interfering with the protein-protein interplay between A*β*-proteins. Given the strong correlation between gut dysfunction and neuropathies, fecal microbiome transplantation (FMT) has been discovered to be a successful treatment approach for restoring a healthy bacterial community inside the gut and has been shown to benefit several diseases, including Alzheimer's. Bacterio-therapy via oral gavage with probiotic supplementation has emerged as a promising potential treatment for neurological illnesses such as Alzheimer's. As a result, the 3xTg murine animal model of AD administered with probiotics early on demonstrated a significant lowering in inflammatory markers and lowered mental impairment, which was correlated with less brain damage and amyloid-beta aggregates accumulation. Furthermore, several research have indicated that probiotic and butyrate administration improve mental health, and memorizing capacity in a D-galactose paradigm of increasing age, a state is known to associate with the development and progress of AD [58].

There are possible mechanisms through which short-chain fatty acids regulate gut and brain connection: SCFAs are the primary metabolic products generated via the intestinal tract microbiome by the anaerobic process of indigestible carbohydrates such as dietary fiber and starch. SCFAs may affect gut-brain connection and cognitive ability, either directly or indirectly. SCFAs are taken by colon cells mostly via sodium- (Na-) dependent monocarboxylate transporters (SMCTs) or H + -dependent monocarboxylate transporters (MCTs) [59]. SCFAs regulate intestinal epithelium immunology, barrier stability, and functionality via coupling to G protein coupled receptors (GPCRs) including free-fatty acid receptors 3 and 2 (FFAR3 & FFAR2), in addition to gpr109A/hcar2 (hydrocarboxylic acid receptor) and gpr164, or via suppressing enzymes like histone protein deacetylases. The interaction of SCFA with the receptors on entero-endocrine cells results in tortuous stimulation of the nervous system via the circulatory system or other pathways, such as vagal, by eliciting the release of insulin and glucagon as well as gamma-aminobutyric acid, also known as serotonin and GABA. Peripherally, SCFAs regulate inflammatory processes primarily by inducing Treg maturation and regulating the production of interleukin Colon-originated SCFAs enter the bloodstream and various other organs, causing activation of brown adipocytes, metabolic function modulation in the hepatocytes, elevated insulin release by beta-pancreatic cells, and energy homeostasis of the whole body. SCFAs can penetrate the BBB through endothelium monocarboxylate carriers and alter BBB stability by upregulating the translation of tight-junction proteins. Finally, SCFAs modulate inflammation in the CNS by influencing glial cell architecture and efficacy, and even by modifying neurotrophic factor production, enhancing neurogenesis, aiding serotonin production, and enhancing neural homeostasis and activity. The association of SCFAs with these gut-brain networks can alter empathy, memory, and the etiology of brain illnesses actively or passively [60].

### 3.9. Mechanisms of Short-Chain Fatty Acid Signal Transduction (Histone Deacetylation Inhibition)

Epigenetic control, which involves chromatin remodeling, DNA methylation, histone modifications, and noncoding RNA regulation, has been illustrated to play a central role in nervous system growth and neurodegenerative disorders such as Amyotrophic lateral sclerosis, Parkinson's disease, Huntington's disease, and Alzheimer's disease. Nucleosomes are the basic building elements of chromosomes, consisting of 147 base pairs of DNA surrounded by double-copy histones (H2B, H2A, H4, and H3). The nucleosome regulates gene expression through two important mechanisms: ATP-dependent chromatin complex remodeling, resulting in fast genomic rearrangements [61]. The ATP-dependent remodeling of histone complexes, which causes rapid chromatin rearrangements, and post-translational alteration of histones at more than 20 possible sites, including acetylation, SUMOylation, methylation, ubiquitylation, and phosphorylation, are the two main mechanisms by which nucleosomes regulate gene expression. Histone acetylation is among the most significant epigenetic mechanisms in the progression of Alzheimer's disease, acting as a link between environmental and hereditary factors [55]. The dynamic activities of two categories of enzymes with contrasting functions, histone deacetylases (HADs), and histone acetyltransferases (HATs) determine the levels of histone acetylation (HDACs). According to their molecular structures, HDAC inhibitors are categorized into four groups: hydroxamates, aliphatic acids, cyclic peptides, and benzamides. SCFAs are derived from aliphatic acid that functions as broad-spectrum inhibitors of HDAC enzymes at mM concentrations. Propionate and butyrate can inhibit noncompetitively class I, class II, and certain class III HDACs. Valeric acid may also be a category I HDAC inhibitor. Butyrate has the greatest inhibitory efficiency of any SCFA, at over 80%; propionate has 60% inhibitory efficiency. Propionate and butyrate induce neutrophil death in the immune system by inhibiting HDAC via a pathway that does not include GPRs or MAPKs. Furthermore, n-butyrate can modulate macrophage functioning in the intestines via suppressing HDACs rather than by triggering toll-like receptor signaling and induction of GPRs [62].

## 4. Revealing the Impact of the Gut on the Neuronal System

The nexus between the brain and gut is generally termed “the gut-brain axis.” The respective axis is presumed to aid in the establishment of cross-talk between the ENS of the GI tract and the CNS of the encephalon. Furthermore, this task is accomplished via the aid of hemoglobin, the vagal/vagus nerve, and passive diffusion [20].

The vagus nerve innervates the viscera via an enormous efferent and afferent network and serves as an intermediary between upper CNS circuits and brain stem parasympathetic control circuitry. It is a multimodal autonomic nerve that originates in the medulla oblongata and travels bilaterally from the brain stem through the neck (where it is coiled with the carotid artery rostrally) and esophagus before diverging peripherally to activate the viscera. The activation and stimulation of the vagus nerve (VNS) can act as a therapeutic alternative to neurodegenerative disorders such as AD, PD, and ALS [63].

The USA Food and Drug Administration (FDA) authorized vagus nerve activation (VNS) for therapeutic application in 1997, and it is an effective and safer adjunctive therapy for refractory various neurological disorders including neurodegeneration. Recent reports discovered that activating the dermatome of the vagus nerve inside the external ear similarly may have a neuronal-protective outcome. Particularly, auricular-vagus nerve stimulation (A-VNS) is the least persistent VNS method that is safest and easiest to conduct, and it is having a parallel restorative impact on VNS. As a result, VNS is particularly equipped for clinical applications. VNS has been shown to successfully decrease postischemic inflammation and oxidative stress, resulting in improved rehabilitative outcomes. In various animal models, VNS-induced neuroprotection is related to the stimulation of central 7-nicotinic acetylcholine receptors. Emerging evidence suggests that 7 7-nicotinic acetylcholine receptors can attenuate inflammatory reactions in Parkinson's disease pathogenesis. Furthermore, 7-nicotinic acetylcholine receptors are engaged in the implementation of murine splenic CD4+ T-cell development. In information on published data on VNS, it was anticipated that VNS could improve inflammation and immunological balance in neurodegenerative disorders [64]. In addition to vagus nerve stimulation, bile moieties are also considered to have a neuroprotective effect against various neurodegenerative diseases. *Ursodeoxycholic acid* and its taurine-conjugate are endogenous water-soluble bile acids with anti-programmed cell death and neuroprotective characteristics, in part by interfering with the mitochondrial mechanisms of apoptosis, hindering the production of unstable free oxygen radicals, limiting the stress of endoplasmic reticulum, and stabilizing the response of misfolded protein, implying a therapeutic potential advantage in neuroapoptosis. Both bile salts are accessible via oral gavage and accessible to the BBB epithelium, penetrating the *in vitro* human neuronal microvascular endothelial cells monolayer, making neurodegenerative illnesses an appealing topic of research. *Ursodeoxycholic acid* and its taurine analog, tauroursodeoxycholic acid (TUDCA), were identified in the parenchyma of the brain of rodents after administration, and in the cerebrospinal fluid of individuals with amyotrophic lateral sclerosis after oral dosage. TUDCA has been shown to have antiapoptotic and therapeutic properties in various experimental models of neurodegenerative ailments. TUDCA's antiapoptotic activity in AD drew huge attention after it was shown that these bile moieties inhibited cell death in primary murine neurons exposed to amyloid peptides [65].

### 4.1. Nitric Oxide: Crusader for Neurodegeneration

Nitric oxide, also referred to as NO, is the chief neurotransmitter of NANC (nonadrenergic and noncholinergic) of the ENS in both animals and humans [66]. The release of this neurotransmitter is induced by glutamate, as it initiates the activation of N-methyl-D-aspartate (NMDA) receptors [22]. Likely sources for NO in the GI tract involve endogenous and penetrating leukocytes, constitutional intestinal tissues, and shriveling of luminal gastric nitrate and nitrite along with denitrifying action by commensal anaerobes contributes to the production of NO. Further studies in the field have revealed that NO from a foreign source mimics native NO in terms of action. Formerly, NO is recognized as a potential relaxing factor derived from the endothelium and is abbreviated as EDRF. Therefore, it mediates the relaxation of a variety of blood vessels [67].

**Figure 4:**
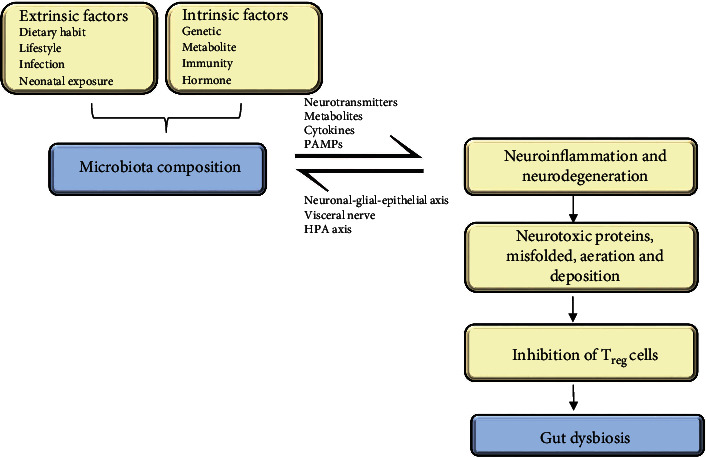
Role of *Escherichia coli* (*E. coli*): Some various ways and genes contribute to the generation of NO within the body of an organism. Among them, *E. coli* serves as a potent medium. Researchers have revealed that *E. coli* is significantly capable of converting nitrites into nitric oxides in a neutral pH environment.

Researchers have revealed that *Escherichia coli (E. coli)* is capable of converting nitrite into NO at pH 7. Moreover, the strain *Klebsiella*, belonging to the Proteobacteria phylum, along with the *Clostridial* strain of the phylum Firmicutes, which forms the vital composition of gut microbiota, have been identified to have the potential to fix nitrogen in the gut of humans [68]. Determination of nitrogen fixation was accomplished by sequencing the metagenomic *nifH* gene in the fecal sample (Figure 4). The NO mechanism ignites the yield of NO and is the principal location for the generation of a substantial quantity of NO concentrations in the gut [68].

NO is a minute, extremely gaseous, diffusible, and responsive molecule that has a less half-life and is processed by an enzyme, nitric oxide synthase (NOS), through the transition of (L) arginine to citrulline through enzymatic action. Various distinguishable genes of NOS are manifested. They are as follows:

1. Neuronal nitric oxide (NOS1, *n*NOS)
2. Inducible nitric oxide (NOS2, *i*NOS)
3. Endothelial nitric oxide (NOS3, *e*NOS)

*n*NOS and *e*NOS produce an nM concentration of the NO molecule and exert an important neuroprotective effect. On the other hand, iNOS produces *μ*M concentrations of NO, which elicits a neuronal toxic response to pro-inflammatory stimulus [69]. In particular, NOS-nitrosylation of the N-methyl-D-aspartate (NMDA) receptor subunit or the caspases at their active sites instructs its neuroprotective function in the regimentation of neuronal cell division, endurance, and distinction [26]. Moreover, it has been revealed through studies that CREB (cyclic AMP-responsive element-binding protein) and Akt protein in brain cells, which are probably granular, are activated by NO. The aforementioned molecules on activation are conditioned to work towards encouraging the endurance of the cell [66].

The other aspect is that a plethora of NO can be extremely harmful and it may be subjected to a redox reaction, which may further lead to the generation of toxic substances such as RNS (reactive nitrogen species). RNS has been identified to possess the potential to render the cell impaired, encourage degeneration of neurons, and induce neurodegenerative maladies [58].

## 5. Conclusion

The review successfully established that the interconnection between the brain and the gut for the generation of neurodegenerative maladies is crucial. Both aerobic and archaebacteria form the basis of the make-up of the gut microbiome. It has been witnessed in the works of the literature discussed above that the fetus when inside the womb is devoid of a microbiome in the gut. Further comprehensive efforts in the field of research towards gut flora have illustrated that the establishment of microbes in the gut is initiated at birth but is completely achieved in the upcoming three years of birth. There are several reasons to which the disturbance of gut-microbiota is linked, but the most potent reason for it is exposure to antibiotics. As the review is directed toward the recognition of the nexus between the microbiome of the gut and its effects on neurodegeneration, both major types of neurodegenerations have been generally discussed to mention the conventional pathways of the generation of the maladies, addition to the disruption of the gut microbiome. Furthermore, NO, which is the chief neurotransmitter for NANC, plays a crucial part in the generation of the ailments. Both Parkinson's and Alzheimer's diseases are linked to the NO pathway. However, it has also been recognized that the source of NO is not only gut-associated microbes but also external environmental and dietary elements that equally contribute to it. Moreover, several genes have been recognized to endogenously produce NO, which further validates that the generation of neurodegeneration has its strings attached to that of the microbiota of the digestive tract. The link between the microbiota of the gut and neurological diseases is generating a rising amount of research interest. A rising body of evidence recommends that the microbiota plays a larger role in the inflection of a variety of pathological and physiological situations, and it is now widely accepted that two-way communications between the microbiota of the gut and brain are required to maintain homeostasis. The neuroendocrine, CNS, neuroimmune systems, enteric nervous system, autonomic nervous system, and microbiota of the colon are all part of the gut-brain axis. Probiotics (living bacteria that are identical to the beneficial microbes present in the human gut) have been shown to help with a range of ailments, including metabolic abnormalities, behavioral issues, and cognitive functioning.
